# Integrated analysis and network pharmacology approaches to explore key genes of Xingnaojing for treatment of Alzheimer's disease

**DOI:** 10.1002/brb3.1610

**Published:** 2020-04-18

**Authors:** Meixia Wang, Shouyong Wang, Yong Li, Gaomei Cai, Min Cao, Lanfang Li

**Affiliations:** ^1^ Department of Pharmaceutical Affiliated Hospital of Jining Medical University Jining China; ^2^ Medication Procurement Office Affiliated Hospital of Jining Medical University Jining China; ^3^ EICU Affiliated Hospital of Jining Medical University Jining China; ^4^ Department of Neurology Ward Affiliated Hospital of Jining Medical University Jining China; ^5^ Continuing Education Office Affiliated Hospital of Jining Medical University Jining China; ^6^ Department of Clinical Pharmacy Affiliated Hospital of Jining Medical University Jining China

**Keywords:** Alzheimer's disease, differentially expressed genes, integrated analysis, network pharmacology

## Abstract

**Background:**

Alzheimer's disease (AD), as a neurodegenerative condition, is one of the leading causes of dementia. Our study aims to explore the key genes of Xingnaojing (XNJ) for treatment of AD by integrated microarray analysis and network pharmacology.

**Methods:**

The differentially expressed genes (DEGs) were identified in AD compared with normal control. According to these DEGs, we performed the functional annotation, protein–protein interaction (PPI) network construction. The network pharmacology was used to explore the potential targets of XNJ in the treatment of AD. The expression level of selected candidate genes was validated by quantitative real‐time polymerase chain reaction (qRT‐PCR).

**Results:**

A total of 1,424 DEGs (620 genes were upregulated and 804 genes were downregulated) between AD and normal control were obtained. The functional annotation results displayed that neuroactive ligand–receptor interaction, regulation of actin cytoskeleton, Estrogen signaling pathway and notch signaling pathway were significantly enriched pathways in AD. Comparing the target genes of four active ingredients, a total of 16 shared genes were found. Among which, HTR2A and ADRA2A were also enriched in pathway of neuroactive ligand–receptor interaction. The expression of 4 DEGs (SORCS3, HTR2A, NEFL, and TAC1) was validated by qRT‐PCR. Except for TAC1, the other 3 DEGs in AD were consistent with our integrated analysis.

**Conclusions:**

The results of this study may provide novel insights into the molecular mechanisms of AD and indicate potential therapeutic targets for AD.

## Graphical abstract

The results of this study may provide novel insights for the molecular mechanisms of Alzheimer's disease (AD) and suggest potential therapeutic targets for AD.

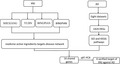

## 1 INTRODUCTION

Alzheimer's disease (AD) is a kind of dementia in aging population. The clinical features of AD mainly include memory loss, cognitive dysfunction behavioral abnormalities, and social disorders (Isaev et al., 2015). At present, the clinical diagnosis of AD requires a variety of examinations such as medical history, neuropsychological evaluation, and various radiological investigations (Yao et al., 2018). However, these diagnostic procedures cannot be used as routine checks for AD because they are time‐consuming. In order to diagnose AD accurately, it is necessary to use biotechnology bioinformatics methods to find disease biomarkers.

With the development of gene expression profiles, bioinformatics comprehensive analysis is the most commonly used ways to find key biomarkers for multiple diseases (Quan et al., 2020; Wang, Wu, Liu, Wu, & Dong, 2015). Nowadays, network pharmacology has become a new topic for us to uncover complex biological processes from the perspective of integrated multi‐component networks (Hopkins, 2008). The holistic approaches of network pharmacology in traditional Chinese medicine research may be viable options for the AD treatment (Jarrell, Gao, Cohen, & Huang, 2018). Xingnaojing (XNJ) is an effective traditional Chinese medicine agents used to treat stroke in China. Network pharmacology studies have reported that XNJ can relieve brain injury and has neuroprotective effects in models of stroke (Ma et al., 2017; Xu et al., 2014). Several previous published in Chinese academic journals have reported the effects of XNJ for AD. AD is thought that the loss of memory because of aggregating beta amyloid (Aβ) and neurofibrillary tangles of hyper‐phosphorylated tau protein (Izadi & Soheilifar, 2018). XNJ exhibits a protective effect against excitatory amino acid toxicity and synaptic plasticity via AKT/mTOR signal pathway in mice with Aβ1‐42‐induced memory deficit. These results provide evidences for the novel and potential application of XNJ for the treatment of AD (Liu, Cao, & Xu, 2019). However, the potential mechanisms of XNJ on AD are not clear, which limits further clinical usage.

In this study, we integrated eight gene expression datasets to obtain the DEGs between AD and normal controls and uncover the molecular mechanisms of AD. Functional enrichment analysis and PPI network were performed to understand the biological functions of these DEGs. The network pharmacology was used to explore the potential mechanisms of XNJ in treating of AD.

## 2 MATERIALS AND METHODS

### 2.1 Microarray data

The gene expression data of AD and normal control used in this study were downloaded from the Gene Expression Omnibus (GEO) database (http://www.ncbi.nlm.gov/geo) with the keywords (Alzheimer's disease) AND “Homo sapiens” [porgn]. Eight datasets (GSE110226, GSE39420, GSE37264, GSE48350, GSE26972, GSE37263, GSE32645, and GSE16759) were selected for datasets base on the selection criteria described as follows: (1) Dataset should be whole‐genome mRNA expression profile by array. (2) Datasets must contain both brain tissue samples of AD and normal control. (3) The datasets should be normalized or original. The impact of different platforms on the sequencing results, we normalized the data through the log function and centralized and standardized the scale function to eliminate the impact of the dimension on the data structure.

### 2.2 Differential expression analysis

MetaMA package was performed to combine data from multiple microarray datasets. Individual P‐values were analyzed and multiple comparison correction false discovery rate (FDR) was obtained according to the Benjamini and Hochberg approach. DEGs were considered with thresholds of FDR < 0.01. The heat map of top 100 DEGs was generated by R package.

### 2.3 Functional enrichment analysis

The David (6.8; https://david.ncifcrf.gov) was used to perform functional enrichment analysis. Gene Ontology (GO) classification and the Kyoto Encyclopedia of Genes and Genomes (KEGG) pathway enrichment analyses were identified as enriched with thresholds of *p* value < .01.

### 2.4 PPI network construction

The PPI network was constructed via Biological General Repository for Interaction Datasets (BioGRID; http://thebiogrid.org/), and then the PPI network was visualized by Cytoscape (3.6.1; http://www.cytoscape.org/). The nodes represent proteins and edges connect the nodes to show their relationship.

### 2.5 Medicine‐active ingredients‐targets‐disease network construction based on network pharmacology

To obtain the medicine‐active ingredients‐targets‐disease network of XNJ, we searched BATMAN‐TCM database, which is the first online Bioinformatics Analysis Tool for molecular mechanism of Traditional Chinese Medicine (http://bionet.ncpsb.org/batman‐tcm/). XNJ was composed of four herbs, such as SHEXIANG, YUJIN, BINGPIAN, and ZHIZI. We input the herb list denoted by ‘SHEXIANG, YUJIN, BINGPIAN and ZHIZI’ with the following default parameters: predicted candidate targets (including known targets) with Score cutoff ≥ 20 and *p*‐values < .05 for each ingredient are presented and used for further bioinformatics analyses. The core idea of this method, first proposed by Perlman, Gottlieb, Atias, Ruppin, and Sharan (2011), is to rank potential drug‐target interactions based on their similarity to the known drug‐target interactions. The targets of XNJ were then obtained by a combination of the targets of SHEXIANG, YUJIN, BINGPIAN, and ZHIZI. The medicine‐active ingredients‐targets‐disease network of XNJ was visualized by Cytoscape (3.6.1). In this network, nodes represented the medicine, active ingredients, targets or disease, and edges represented the interactions of them.

### 2.6 Quantitative real‐time polymerase chain reaction (qRT‐PCR) confirmation

Based on the results of GEO integration analysis and network pharmacology, four genes (SORCS3, HTR2A, NEFL, and TAC1) were selected as candidate genes. Eleven blood samples from six AD patients and five healthy individuals were obtained. All subjects were first on an empty stomach for 12 hr, and we collected the blood samples by venipuncture at 7:00–8:00 of the next morning. This study has been approved by the ethics institute of our hospital. The signed informed consents of all the participants were obtained.

Total RNA was isolated using RNA simple total RNA kit (Invitrogen). Fast Quant RT Kit (Invitrogen) was utilized to obtain the complementary DNA. With Super Real PreMix Plus SYBR Green (Invitrogen), quantitative real‐time PCR was generated using the ABI 7500 system. The amplification process was performed under the following conditions: 15 min at 95°C followed by 40 cycles of 10 s at 95°C, 30 s at 55°C, 32 s at 72°C, and 15 s at 95°C, 60 s at 60°C, 15 s extension at 95°C. The 2^−ΔΔ^ *^Ct^* method was used to address the data. The human ACTB was used as endogenous controls for gene expression.

## 3 RESULTS

### 3.1 DEGs in AD

**FIGURE 1:**
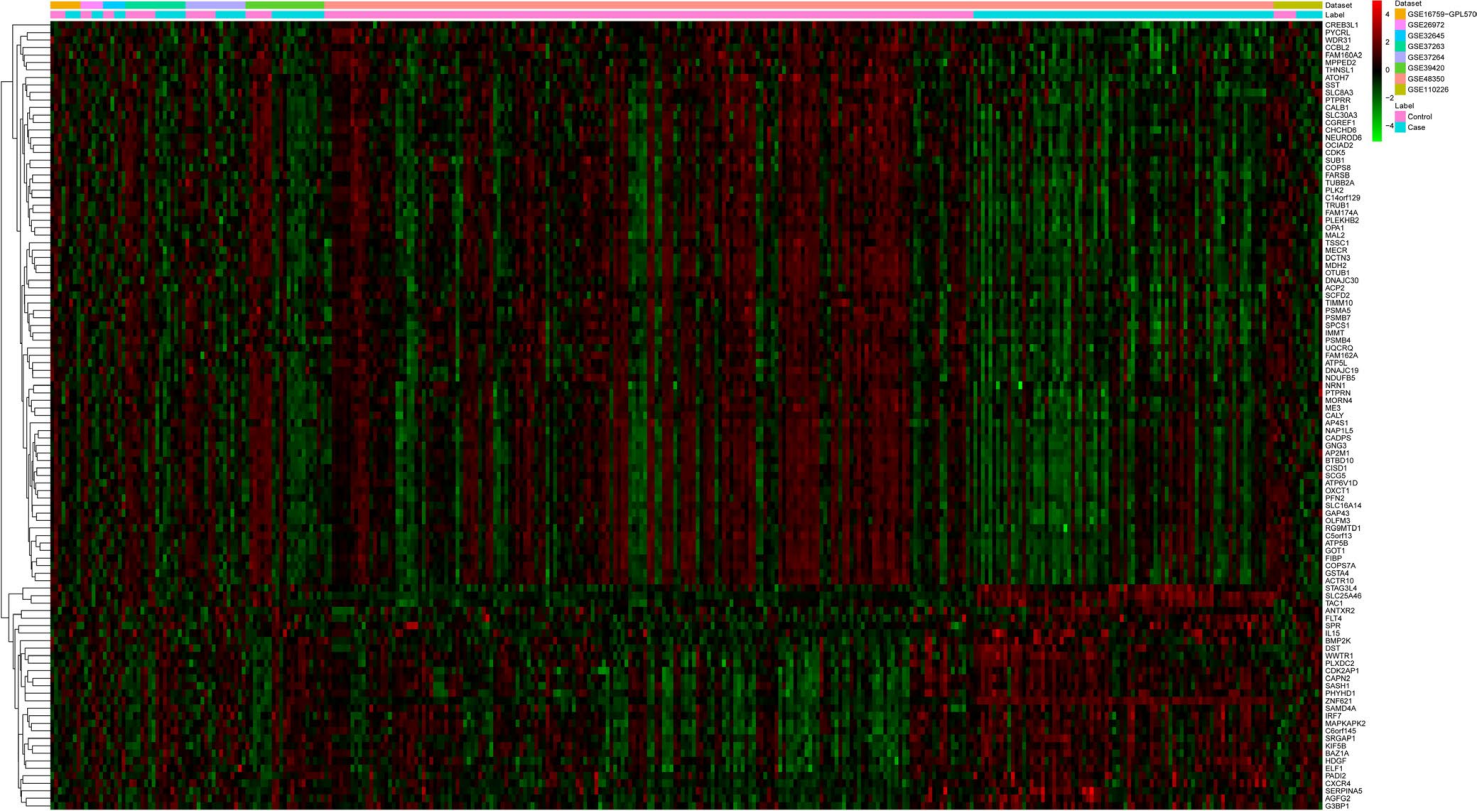
The heat map of top 100 DEGs in AD compared with normal control. Row and column represented DEGs and GEO data, respectively. The color scale represented the expression levels

Eight datasets (GSE110226, GSE39420, GSE37264, GSE48350, GSE26972, GSE37263, GSE32645, and GSE16759) were enrolled from GEO (Table 1). Samples of GSE110226, GSE39420, GSE37264, GSE48350, GSE26972, GSE37263, GSE32645, and GSE16759 were obtained from participants of USA, Spain, Singapore, USA, Israel, Singapore, Austria, and USA, respectively. Compared with the healthy controls, 1,424 DEGs (620 genes were upregulated and 804 genes were downregulated) in AD were obtained with FDR < 0.01. The heat map of top 100 DEGs in AD versus normal control was manifested in Figure 1.

**TABLE 1:** Gene expression datasets used in this study

GEO accession	Author	Platform	Samples (N:P)	Year	Tissue
GSE110226	Edward G Stopa	GPL10379 Rosetta/Merck Human RSTA Custom Affymetrix 2.0 microarray [HuRSTA‐2a520709]	6:7	2018	Choroid plexus tissue
GSE39420	Anna Antonell	GPL11532 [HuGene‐1_1‐st] Affymetrix Human Gene 1.1 ST Array [transcript (gene) version]	7:7	2015	Brain tissue
GSE37264	Michelle GK Tan	GPL5188 [HuEx‐1_0‐st] Affymetrix Human Exon 1.0 ST Array [probe set (exon) version]	8:8	2014	Brain tissue
GSE48350	Nicole Claudia Berchtold	GPL570 [HG‐U133_Plus_2] Affymetrix Human Genome U133 Plus 2.0 Array	173:80	2014	Brain tissue
GSE26972	Amit Berson	GPL5188 [HuEx‐1_0‐st] Affymetrix Human Exon 1.0 ST Array [probe set (exon) version]	3:3	2012	Entorhinal cortex tissue
GSE37263	Michelle GK Tan	GPL5175 [HuEx‐1_0‐st] Affymetrix Human Exon 1.0 ST Array [transcript (gene) version]	8:8	2012	Brain tissue
GSE32645	Isabella Wimmer	GPL4133 Agilent‐014850 Whole Human Genome Microarray 4x44K G4112F (Feature Number version)	3:3	2011	Cortex tissue
GSE16759	Juan Nunez‐Iglesias	GPL570 [HG‐U133_Plus_2] Affymetrix Human Genome U133 Plus 2.0 Array	4:4	2011	Parietal lobe tissue

### 3.2 Functional enrichment analysis

**FIGURE 2:**
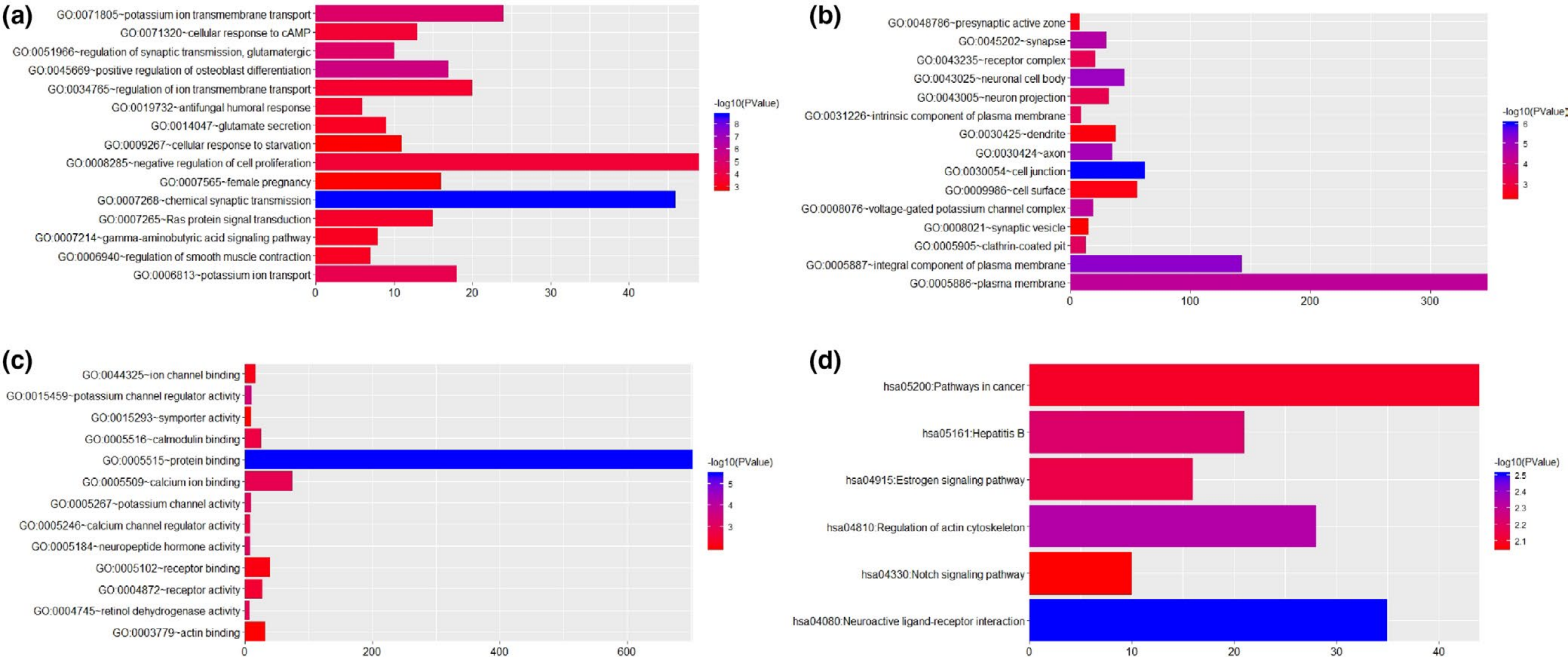
The top 15 most significantly enriched GO terms of DEGs in AD compared with normal control. The x‐axis shows ‐log P and y‐axis shows GO terms and KEGG pathways. (a) Biological process. (b) Molecular function. (c) Cellular component. (d) KEGG pathways

GO enrichment analysis and KEGG pathways analysis were performed to obtain a deeper insight into the biological functions and pathways of DEGs selected in this study. As shown in Figure 2a–c, chemical synaptic transmission (*p* = 1.95E−09), potassium ion transmembrane transport (*p* = 1.35E−05), cell junction (*p* = 9.19E−07), neuronal cell body (*p* = 8.45E−06), protein binding (*p* = 3.45E−06), and neuropeptide hormone activity (*p* = 9.28E−04) were significantly enriched GO terms. As shown in Figure 2d, total 6 KEGG pathways were mainly enriched in pathway of neuroactive ligand‐receptor interaction (*p* = .003091196), regulation of actin cytoskeleton (*p* = .004541129), estrogen signaling pathway (*p* = .007003958), and notch signaling pathway (*p* = .008787427).

### 3.3 PPI network

**FIGURE 3:**
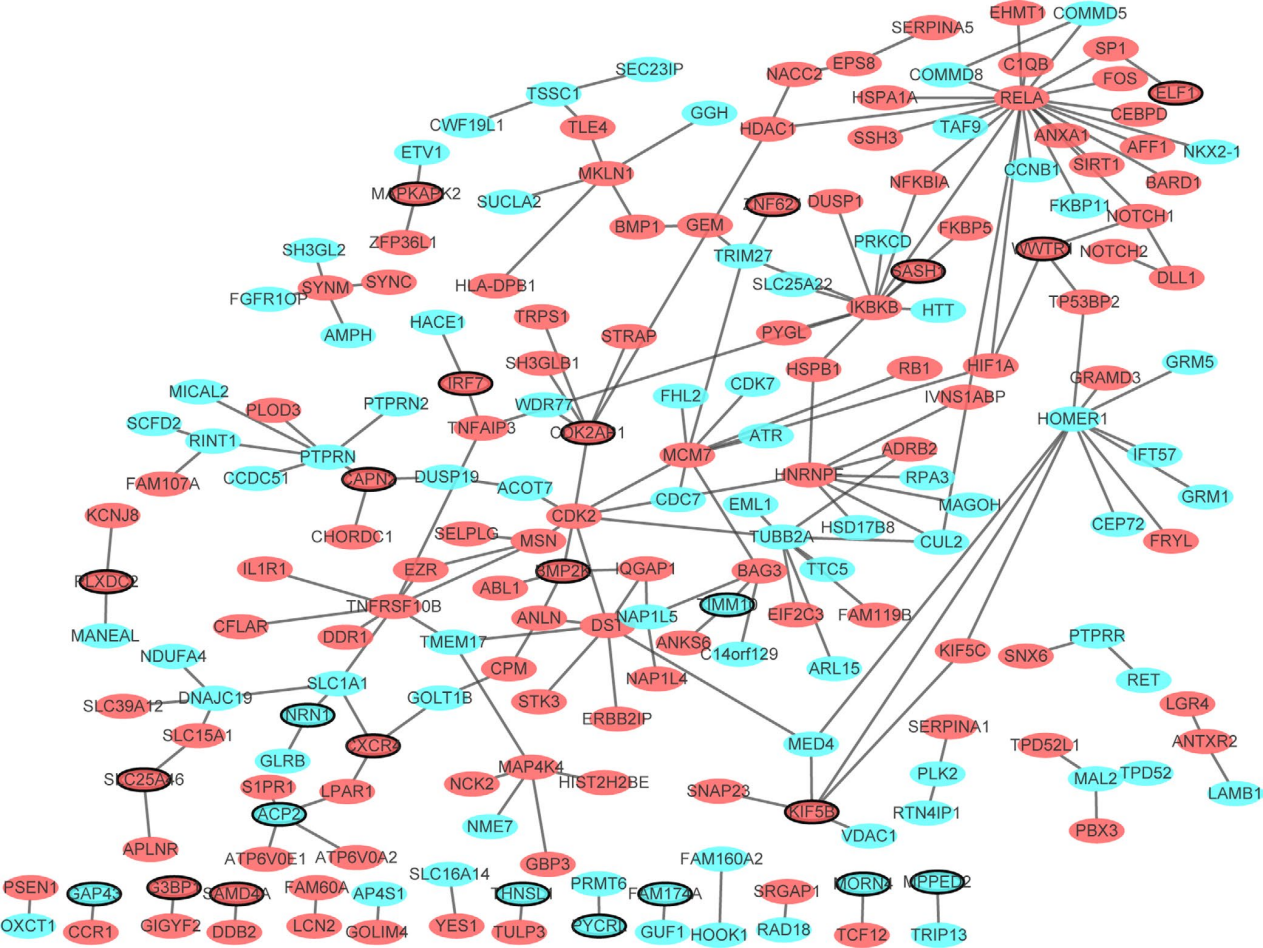
PPI network. Ellipses were used to represent nodes, and lines were used to represent edges. Green represents a downward adjustment, and red represents a downward adjustment. The black border indicates top 20 Up/Down

The PPI network of top 100 DEGs in AD was consisted of 198 nodes and 195 edges (Figure 3). RELA (degree = 23), IKBKB (degree = 12), HOMER1 (degree = 10), MCM7 (degree = 9), HNRNPF (degree = 8), CDK2 (degree = 8), TNFRSF10B (degree = 8), TUBB2A (degree = 8), DST (degree = 7), CDK2AP1 (degree = 6), PTPRN (degree = 6), MAP4K4 (degree = 5), MKLN1 (degree = 5), KIF5B (degree = 5), and ACP2 (degree = 4) were considered the top 15 DEGs with high degree.

### 3.4 Medicine‐active ingredients‐targets‐disease network

**FIGURE 4:**
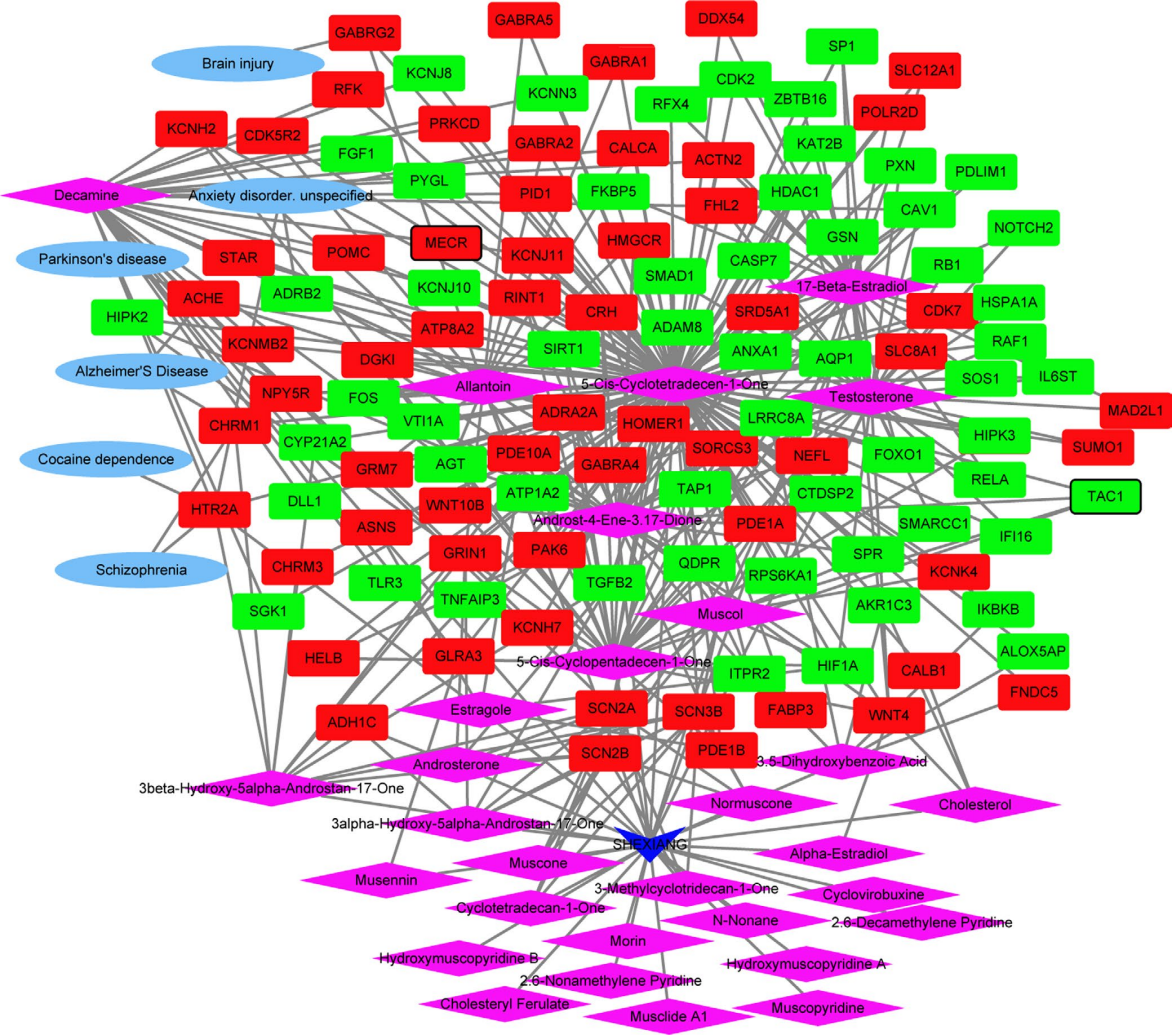
The medicine‐active ingredients‐targets‐disease network of SHEXIANG. The inverted triangles, rhombus, ellipses, and rectangles represent the composition of traditional Chinese medicine, active ingredients, disease, and DEGs. Red and blue colors indicate upregulated and downregulated, respectively. The black border indicates top 10 Up/Down

**FIGURE 5:**
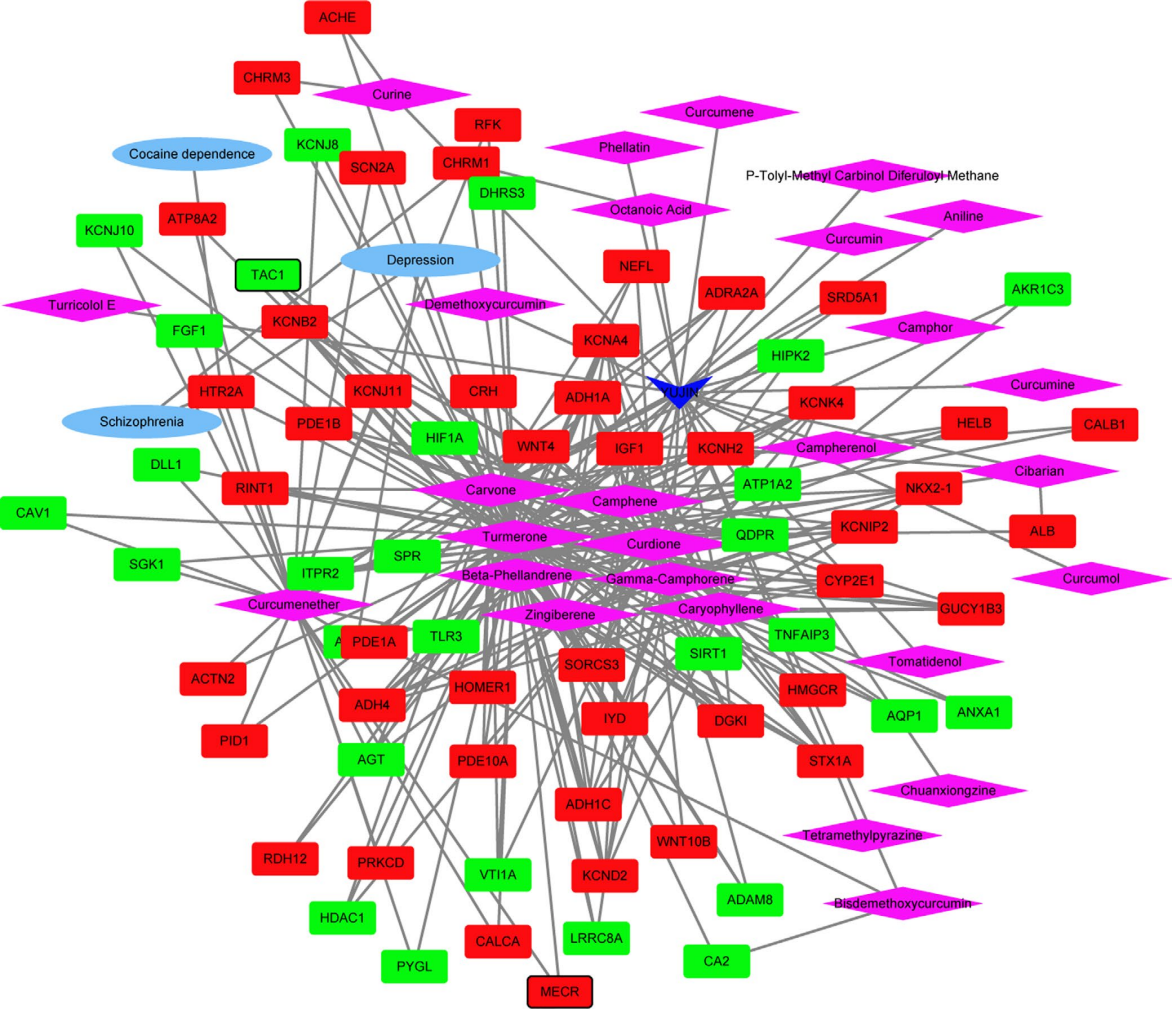
The medicine‐active ingredients‐targets‐disease network of YUJIN. The inverted triangles, rhombus, ellipses, and rectangles represent the composition of traditional Chinese medicine, active ingredients, disease, and DEGs. Red and blue colors indicate upregulated and downregulated, respectively. The black border indicates top 10 Up/Down

**FIGURE 6:**
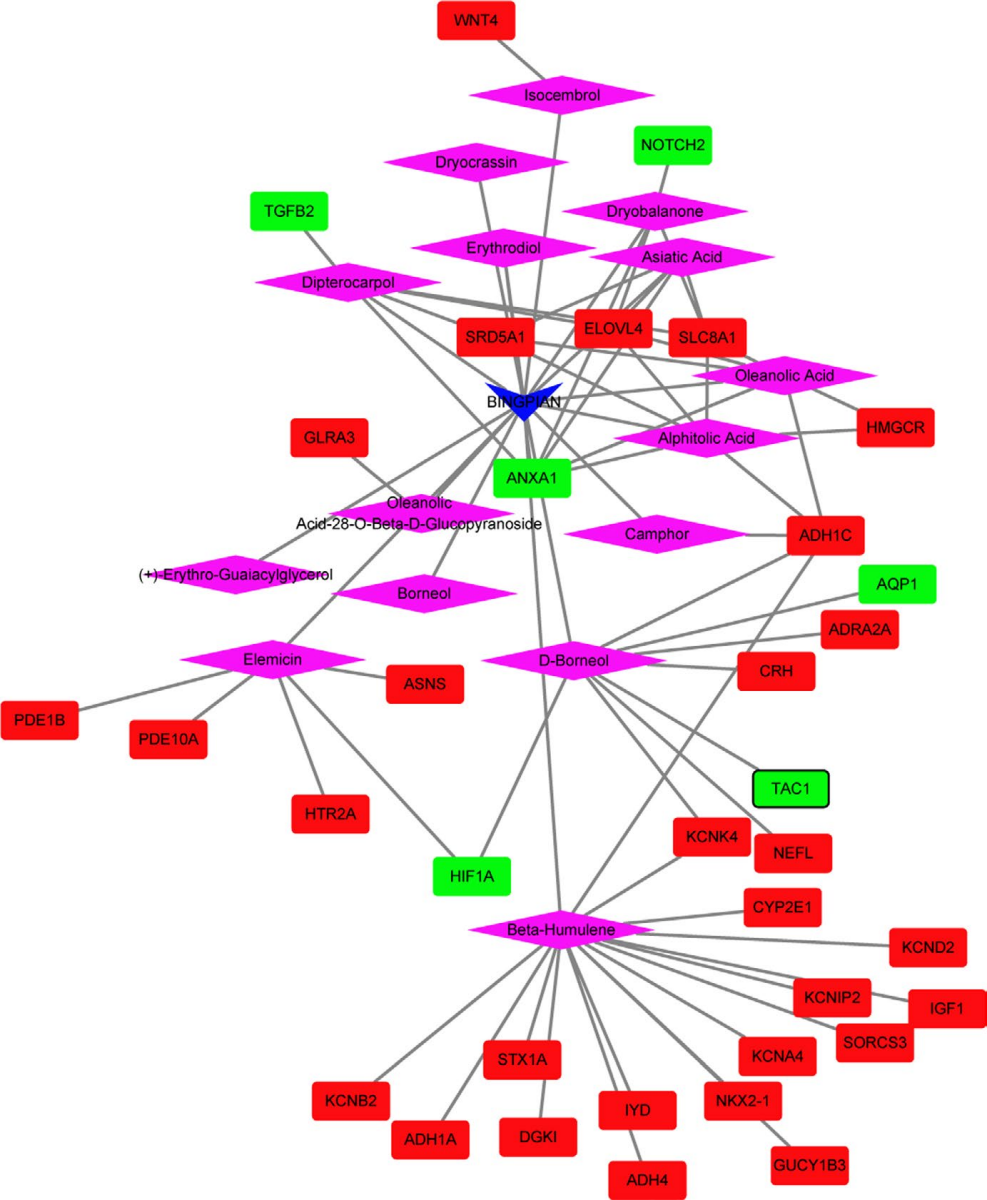
The medicine‐active ingredients‐targets‐disease network of BINGPIAN. The inverted triangles, rhombus, ellipses, and rectangles represent the composition of traditional Chinese medicine, active ingredients, disease, and DEGs. Red and blue colors indicate upregulated and downregulated, respectively. The black border indicates top 10 Up/Down

**FIGURE 7:**
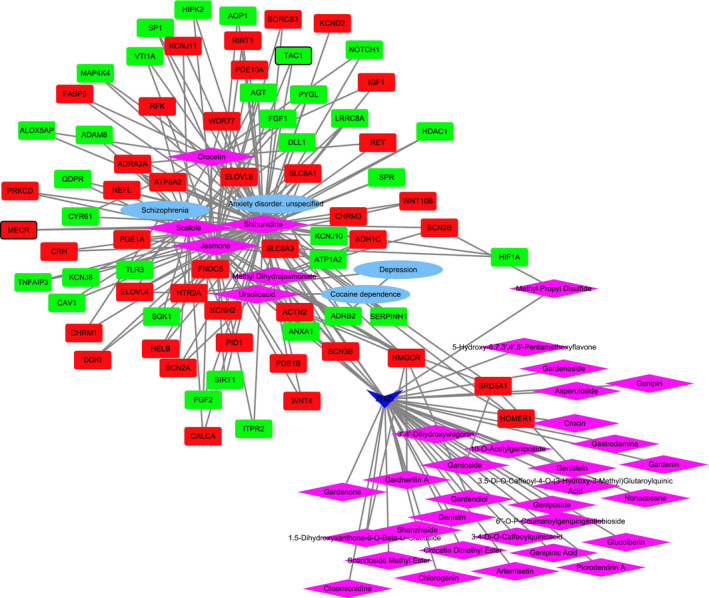
The medicine‐active ingredients‐targets‐disease network of ZHIZI. The inverted triangles, rhombus, ellipses, and rectangles represent the composition of traditional chinese medicine, active ingredients, disease, and DEGs. Red and blue colors indicate upregulated and downregulated, respectively. The black border indicates top 10 Up/Down

**FIGURE 8:**
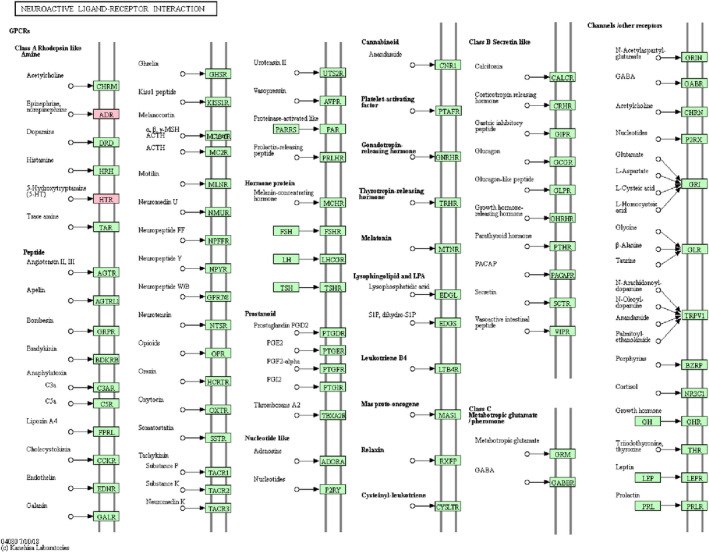
The neuroactive ligand–receptor interaction pathway. The red rectangles were represented the components regulated by the DEGs that enriched in AD

The medicine‐active ingredients‐targets‐disease network of SHEXIANG was consisted of 155 nodes and 389 edges (Figure 4). SHEXIANG's active ingredient 5‐Cis‐Cyclotetradecen‐1‐One and Allantoin may involve in the brain injury process by regulating GABRG2. SHEXIANG's active ingredient 5‐Cis‐Cyclotetradecen‐1‐One and Allantoin may play an important role in the pathogenesis of Parkinson's disease and Alzheimer's disease by regulating ACHE. The medicine‐active ingredients‐targets‐disease network of YUJIN was consisted of 105 nodes and 283 edges (Figure 5). The medicine‐active ingredients‐targets‐disease network of BINGPIAN was consisted of 52 nodes and 73 edges (Figure 6). The medicine‐active ingredients‐targets‐disease network of ZHIZI was consisted of 115 nodes and 205 edges (Figure 7). Comparing the target genes of four active ingredients (SHEXIANG, YUJIN, BINGPIAN, and ZHIZI), a total of 16 shared genes were found. Among which, HTR2A and ADRA2A were also enriched in pathway of neuroactive ligand–receptor interaction (Figure 8).

### 3.5 qRT‐PCR confirmation

**FIGURE 9:**
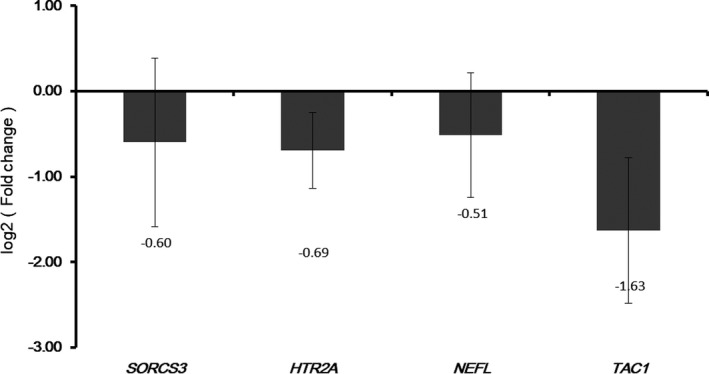
The validation of the expression levels of selected DEGs in AD. The *x*‐axis shows DEGs, and *y*‐axis shows log2 (fold change) between AD and normal controls

To verify the results of integration analysis, we measured the expression of candidate genes (SORCS3, HTR2A, NEFL, and TAC1) using the qRT‐PCR (Figure 9). SORCS3, HTR2A, NEFL, and TAC1 were members of 16 shared genes in target genes of four active ingredients (SHEXIANG, YUJIN, BINGPIAN, and ZHIZI). Compared with normal control, SORCS3, HTR2A, and NEFL were downregulated in AD in the qRT‐PCR confirmation which was consistent with that in integration analysis. Compared with normal control, TAC1 was downregulated in AD in qRT‐PCR confirmation while upregulated in AD in integration analysis. Overall, most of the qRT‐PCR results were consistent with the results of integration analysis.

## 4 DISCUSSION

AD, as a neurodegenerative condition, is one of the leading causes of dementia. Early diagnosis of AD is an urgent issues in discovery and treatment of AD. To uncover key genes and related pathways of AD, we downloaded the GSE110226, GSE39420, GSE37264, GSE48350, GSE26972, GSE37263, GSE32645, and GSE16759 datasets from the GEO database to obtain gene expression data from AD patients. Based on the previously published profiles, we firstly obtained the DEGs for AD. Furthermore, we performed functional enrichment analysis and PPI network on these genes to predict AD‐related genes and biological processes. Finally, the network pharmacology was used to explore the potential mechanism of XNJ in treating of AD.

Sortilin‐related VPS10 domain containing receptor 3 (SORCS3) is a member of the vacuolar protein sorting 10 receptor family and expressed in the brain. Multiple evidences have suggested that SORCS3 is considered to be a major genetic risk factor for AD (Reitz, 2012, 2015). Hermey et al. (2019) found that the level of SORCS3 is downregulated in the frontal cerebral cortex of AD mouse model. SORCS3 is reduced in AD compared with control brains and is associated with increased risk of AD (Reitz et al., 2013). One study displayed that SORCS3 is also a risk gene for major depressive disorder (Ni et al., 2018). Intriguingly, single nucleotide polymorphisms of SORCS1, a family member of SORCS3, is associated with AD susceptibility (Reitz et al., 2011). In this study, SORCS3 was downregulated in AD in qRT‐PCR confirmation and integration analysis. The results of network pharmacology showed that SORCS3 was one of 16 shared genes in target genes of four active ingredients of XNJ. These finds further confirmed that SORCS3 might be involved in AD.

Serotonin receptor 2A (HTR2A), a neurotransmitter with multiple functions, which codes for the serotonin receptor type 2A. HTR2A has been associated with selective serotonin reuptake inhibitors response in depressed patients (Quesseveur et al., 2013). Preclinical studies in humans provided support for the involvement of HTR2A in major depressive disorder (Fabbri, Marsano, & Serretti, 2013). Polymorphisms of HTR2A may be associated with the efficacy of antidepressants in the MDD therapy (Lin, Jiang, Kan, & Chu, 2014). The serotonin receptor type 2A receptors are downregulated in frontal and temporal cortical of AD patients (Lai et al., 2005). Polymorphism of serotonin receptor type 2A receptor may be associated with expression of agitation/attack in AD patients (Gotovac, Nikolac Perković, Pivac, & Borovečki, 2016; Ramanathan & Glatt, 2009). Fehér et al. (2013) reported that polymorphism of HTR2A has no influence for AD, but polymorphisms of the serotonin transporter and HTR2A for possible association with AD. The formula Tian‐Ma‐Gou‐Teng‐Yin inhibits the progression of AD by regulating key target gene HTR2A (Wang et al., 2018). In the study, HTR2A was reduced in AD in qRT‐PCR confirmation and integration analysis. HTR2A was enriched in pathway of neuroactive ligand–receptor interaction. Therefore, we speculated that HTR2A may be involved in the progress of AD by regulating pathway of neuroactive ligand‐receptor interaction.

Neurofilament light (NEFL) is a putative marker of neurodegeneration‐related axonal injury (Zetterberg et al., 2006). Serum NEFL concentration is correlated with measures of familial Alzheimer disease stage and severity, indicating that serum NEFL level may be a viable biomarker of early AD‐related neurodegeneration (Weston et al., 2017). Zhou et al. found suggested that plasma NEFL levels may not be a useful biomarker for the diagnosis of AD (Zhou et al., 2017). A recent study showed that NEFL dynamics in serum predict AD progression and brain neurodegeneration at the early presymptomatic stages (Preische et al., 2019). Here, NEFL was one of the DEG between AD and normal tissue. This finding further provides evidence indicated that NEFL may be biomarker of AD‐related neurodegeneration.

## 5 CONCLUSION

We obtained 1,424 DEGs between AD and normal tissue base on the GEO datasets. KEGG pathways analysis displayed that the pathway of neuroactive ligand–receptor interaction was closely associated with AD. A total 16 common target genes in SHEXIANG, YUJIN, BINGPIAN, and ZHIZI active ingredients. These 16 genes may have important research value in the treatment of AD by XNJ. Interestingly, HTR2A and ADRA2A were members of 16 common target genes and also enriched in pathway of neuroactive ligand–receptor interaction. However, this study has several limitations that need to be acknowledged. The number of samples for qRT‐PCR confirmation was small. More samples are needed to validate expression of pivotal DEGs. In addition, in model systems or cell lines, experiments are necessary to uncover the biological functions of key DEGs in AD in future studies.

## CONFLICT OF INTEREST

The authors declare that they have no conflict of interest.

## AUTHOR CONTRIBUTIONS

Meixia Wang contributed to the conception of the study. Shouyong Wang and Yong Li contributed the materials and performed the experiment. Gaomei Cai, Min Cao and Lanfang Li performed the data analyses. Meixia Wang contributed significantly in writing the manuscript. All authors read and approved the final manuscript.

## Data Availability

The dataset supporting the conclusions of this article is included within the article.
